# Geropalliative Caring Model analysis and assessment according to Fawcett’s criteria

**DOI:** 10.1590/1980-220X-REEUSP-2023-0288en

**Published:** 2024-02-23

**Authors:** Rudval Souza Silva, Larissa Coelho Barbosa, Marcos Antônio Gomes Brandão, Raércia dos Santos Carneiro, Nuno Damácio de Carvalho Félix, Manuela Bastos Alves

**Affiliations:** 1 Universidade do Estado da Bahia, Colegiado de Enfermagem, Senhor do Bonfim, BA, Brazil.; 2 Universidade Federal da Bahia, Programa de Pós-graduação em Enfermagem e Saúde, Salvador, BA, Brazil.; 3 Universidade Federal do Rio de Janeiro, Escola de Enfermagem Anna Nery, Rio de Janeiro, RJ, Brazil.; 4 Universidade Federal do Recôncavo da Bahia, Colegiado de Enfermagem, Santo Antônio de Jesus, BA, Brazil.

**Keywords:** Hospice and Palliative Care Nursing, Aged, Nursing Process, Nursing Theory, Evaluation Study, Enfermería de Cuidados Paliativos al Final de la Vida, Anciano, Proceso de Enfermería, Teoría de Enfermería, Estudio de Evaluación, Enfermagem de Cuidados Paliativos na Terminalidade da Vida, Idoso, Processo de Enfermagem, Teoria de Enfermagem, Estudo de Avaliação

## Abstract

**Objectives:**

To analyze and assess Lee Geropaliative Caring Model according to Fawcett’s criteria.

**Method:**

A theoretical-reflective study about the Geropaliative Caring Model. The analysis resulted in a detailed review of the scope, context and content of a situation-specific theory, in order to determine aspects related to nursing practice and research, and assessment addressed the concrete concepts developed in theory, their significance, internal consistency, parsimony, testability and empirical and pragmatic adequacy in nursing as a discipline.

**Results:**

Based on the analysis, a situation-specific theory was used based on the science of care and aimed at caring for older adults undergoing palliation and their families, structured into four fields, namely: (1) aligning care; (2) keeping safe; (3) comforting body/mind/spirit; and (4) facilitating transitions.

**Final considerations:**

The analyzed and assessed theory leads to a structure that includes well-defined, delimited and interrelated concepts, based on the science of care as a grand theory that allowed Geropaliative Care Model derivation.

## RESUMO

**Objetivo:**

Analisar e avaliar o Modelo de Cuidados Geropaliativos de Lee segundo os critérios de Fawcett.

**Método:**

Estudo teórico-reflexivo acerca do Modelo de Cuidados Geropaliativos. A análise suscitou em uma revisão detalhada do escopo, contexto e conteúdo de uma Teoria de Situação Específica, a fim de determinar os aspectos relacionados à prática e pesquisa na enfermagem, e a avaliação abordou os conceitos concretos desenvolvidos na teoria, sua significância, consistência interna, parcimônia, testabilidade e adequação empírica e pragmática na enfermagem enquanto disciplina.

**Resultados:**

A partir da análise, utilizou-se uma Teoria de Situação Específica baseada na ciência do cuidar e direcionada aos cuidados à pessoa idosa em paliação e sua família, estruturada em quatro domínios, a saber: (1) alinhamento dos cuidados; (2) manutenção da segurança; (3) conforto corpo/mente/espírito; e (4) facilitação dos processos de transições.

**Considerações finais:**

A teoria analisada e avaliada direciona para uma estrutura que contempla conceitos bem definidos, delimitados e inter-relacionados, com base na ciência do cuidar enquanto grande teoria que permitiu a derivação do Modelo de Cuidados Geropaliativos.

## RESUMEN

**Objetivo:**

Analizar y evaluar el Modelo de Atención Geropaliativa de Lee según los criterios de Fawcett.

**Método:**

Estudio teórico-reflexivo sobre el Modelo de Atención Geropaliativa. El análisis resultó en una revisión detallada del alcance, contexto y contenido de una Teoría de Situaciones Específicas, con el fin de determinar aspectos relacionados con la práctica y la investigación de la enfermería, y la evaluación abordó los conceptos concretos desarrollados en la teoría, su significado, consistencia interna, parsimonia, contrastabilidad y adecuación empírica y pragmática en la enfermería como disciplina.

**Resultados:**

A partir del análisis, se utilizó una Teoría de Situaciones Específicas basada en la ciencia del cuidado y orientada al cuidado de las personas mayores en paliación y de sus familias, estructurada en cuatro dominios, a saber: (1) alineación del cuidado; (2) mantener la seguridad; (3) comodidad cuerpo/mente/espíritu; y (4) facilitación de los procesos de transición.

**Consideraciones finales:**

La teoría analizada y evaluada conduce a una estructura que incluye conceptos bien definidos, delimitados e interrelacionados, basándose en la ciencia del cuidado como una gran teoría que permitió derivar el Modelo de Cuidado Geropaliativo.

## INTRODUCTION

From the perspective of the World Health Organization, palliative care (PC) aims to provide a comprehensive and active care approach to individuals of all ages who are experiencing intense suffering related to their health, resulting from a serious and chronic illness, especially those at the end of life, with the core of improving the quality of life of patients, their families and their caregivers^(1)^.

Considering the aging of the world population, actions related to PC are becoming increasingly necessary. Each year, around 22 million older adults aged 70 and older need this care without, however, having access and, of these, 78% live in low- income countries^(2)^. Therefore, PC provision remains a constant challenge for health systems.

Care needs of older adults undergoing palliation are no different from other populations (children, young people and adults), however they have particularities related to the phenomenon of aging or critical attributes of geriatric PC, which are: vulnerability in pain control; unpredictable disease trajectories; geriatric syndromes; chronic conditions and comorbidities; reduction of social support networks; limitations of health insurance; multiple contexts in transition to aging; risk of ineffective communication; and situations typical of aging that are independent of the disease^(3)^. Therefore, given the unprecedented growth of population aging, attention to critical attributes related to geriatric PC is urgent and necessary.

The literature is full of PC models for different contexts and populations, such as for more focused situations such as emergencies^(4)^, situations of clinical conditions such as heart failure^(5)^, with models for specific population segments or for all users with PC needs at national levels^(6,7)^. Such proposals conform to models or structures of care, generally not assuming a theoretical configuration or having their focus established from the perspective of the nursing discipline.

In 2018, American nurse Susan M. Lee proposed that the Lee Geropalliative Caring Model be considered a situation-specific theory (SST). From Lee’s perspective, the aforementioned theory would guide the nursing team’s practice in caring for older adults in their last one to two years of life^(8)^. The author recognizes that older adults are more vulnerable to chronic diseases at an advanced step, such as cancer, cardiorespiratory diseases or dementia^(3)^. Therefore, they are more susceptible to PC needs. In general, SST is based on philosophy and science of care assumptions^(9)^, using a fundamental perspective of providing well-being among older adults based on subjective assessment that leads persons to perceive life in a positive way and with hope for the future^(3)^.

The basis of the Lee Geropalliative Caring Model applies elements of a grand nursing theory, Dr. Jean Watson’s Theory of Human Caring^(9)^, as a theoretical structure that addresses generalized phenomenological aspects of nursing, honoring autonomy, choices and meanings for the person based on a deep connection between patient, professional and family, with intentionality, compassionate listening and well-being promotion.

Given this scenario, the relevance of planning actions to provide older adults in PC and their families with a less painful experience emerges, in addition to understanding their needs and attitudes, in order to respond to them, paying attention to the sociocultural context in which they are inserted. Family members, in turn, need to respect and be sensitive to PC patients’ pain, maintaining their individuality and dignity^(10)^.

Because it is classified as an SST, the Lee Geropalliative Caring Model has facilitated the potential for empirical use, encompassing a topic of great social impact. However, its publication is recent and dissemination through publications is still scarce, which calls for theoretical assessment. The theorist herself states that, even though the model has been assessed in practice for seven years by more than five hundred nurses, she still considers a formal theoretical assessment to be necessary^(8)^.

Theory assessment as a metatheoretical, structured and formal strategy can help determine which theory is most appropriate for use in nursing research and practice, allowing to compare and contrast different explanations for the same nursing phenomenon^(11)^. Furthermore, the assessment process can be considered a type of internal validation, focusing on determining its suitability for use and verifying the epistemological approach^(12)^. Therefore, from a scientific point of view, an assessment using structured criteria can be a useful strategy to anticipate the empirical test step.

Therefore, the need to theoretically assess the Lee Geropalliative Caring Model is assumed to provide useful evidence of the generalization capacity of its concepts and statements, particularly because practice induction elements that constituted the theory were only obtained in health care in North American hospitals^(8)^, which requires assessing the nature of application to different populations and contexts.

Given these considerations, this study aimed to analyze and assess the Lee Geropalliative Caring Model according to Fawcett’s criteria.

## METHOD

This is a theoretical-reflexive study, developed in the first semester of 2023, resulting from doctoral research carried out at a public university in northeastern Brazil. The study assesses the Geropalliative Caring Model proposed by Suzan Lee^(3,8,13)^, an SST whose particularity is end-of-life care for older adults.

The analysis and assessment of the aforementioned SST were supported by criteria proposed by Jacqueline Fawcett^(14)^, whose application enabled the development of a descriptive, analytical and critical view of the phenomenon in question.

The analysis step required a detailed review of all available primary sources accessible to the research team to verify scope, content and context criteria^(3,8,13,15,16)^. Thus, the goal was to determine aspects related to nursing practice and research for an objective and non-judgmental description of the theory, carrying out a systematic examination of exactly what the author wrote about the theory, its scope, context and content^(14)^.

The assessment step required judgments about how SST meets the significance, internal consistency, parsimony, testability, empirical adequacy and pragmatic adequacy criteria^(14)^, in order to provide a theoretical framework to contribute to reflections on the theory and its intrinsic aspects, such as its influence, significance, internal consistency, parsimony, testability, and empirical and pragmatic suitability for the nursing discipline^(14)^.

This research method, with the steps of theory analysis and assessment, aims to strengthen nursing practices and confirm its usefulness, applicability and value in nursing education, practice and research. In this regard, studies^(17,18)^ that have used this strategy to analyze and assess nursing theories deserve to be highlighted.

## RESULTS AND REFLECTION

Compared to medium-range theories, SST are much less abstract and can bring into their structures a level of abstraction comparable to that of professional practice documentation models, such as nursing records or care plans. Usually, an SST aims to answer a set of coherent questions about a given situation limited to a scope and focus^(19)^. They are oriented towards a specific nursing phenomenon that reflects clinical practice, peculiar to a particular population or field of practice.

SST are historically and socially contextualized, developed to incorporate rather than transcend time or social and political structures. Although limited in scope and content, its context is comprehensive^(20)^. Its greatest development and publications based on the derivation and deduction of grand theories or medium-range theories occurred in the last decade of the 20^th^ century and the first of the 21^st^ century^(21)^.

From the analysis, we verified that the scope of the Lee Geropalliative Caring Model, while an operational model with more accessible application in clinical situations, is aimed at PC, focused on older adults in end-of-life care. It was derived from the Theory of Human Caring^(9)^, which is a major nursing theory and the first to support nursing practice in PC^(8)^.

Although called the “Lee Geropalliative Caring Model”, the theorist considers it an SST due to its low level of abstraction, because it reflects the specific phenomenon of nursing care in the context of limited life expectancy and because it is linked to practice and limited in generalization^(8)^. Still in scope, it presents itself as a descriptive theory^(14)^ that addresses the specific needs of older adults with late-stage, life-threatening chronic diseases, such as cancer, dementia and frailty who would benefit from approach to PC, more specifically end-of-life care, from care promotion that has well-being at its core in the context of aging. To this end, an interactive relationship between nurses, older adults and their family is essential. It is characterized by a care model aimed at increasing the effectiveness of nurses’ actions towards achieving well-being^(3,8,9)^.

An SST becomes, due to its properties, more operational and applicable in clinical situations, usually having a lower level of abstraction when compared to medium-range theories. This type of theory was proposed by nurses and theorists Eun Ok Im and Afaf Ibraim Meleis in 1999 in response to the limitations of theorizing with a broader scope, in particular the excessive reach of generalization incorporated in grand and medium-range theories^(19)^.

Its content and context are guided by dealing with end-of-life care. End-of-life care is understood as an approach to PC that older adults must receive during the last days of their life, from the moment it becomes clear that they are in a state of progressive and inexorable decline, approaching death^(22)^. In this context, nurses have a prominent role in planning and implementing nursing actions/interventions aimed at physical and emotional well-being as well as at the independence of older adults in carrying out activities of daily living whenever possible, and should, for both, document their practice based on theoretical support in the Nursing Process and the use of Standardized Language Systems^(23)^.

Regarding content, the criteria proposal described by Jacqueline Fawcett^(14)^ requires to identify concepts and propositions of the nursing metaparadigm addressed by the theory, in addition to the basic philosophical claims, the conceptual model of theoretical derivation and the contributions of nursing knowledge and circumscribed to the theory.

Thus, the Geropalliative Caring Model derives from the Theory of Human Caring^(9)^, which can be considered the conceptual model of derivation and the supplier of its philosophical claims. The Theory of Human Caring has as its central aspects the Clinical Caritas Process, composed of 10 elements based on supporting persons under care with a strong predictor of well-being that should be the result to be achieved by patient and family based on the care provided by nurses.

To this end, some indicators are notable in achieving patient and family well-being, such as the expressed desire to maintain patient/family/nurse interaction and promotion of actions that provide comfort and well-being based on harmony with patients’ future prospects so that it is possible to create a therapeutic environment (healing) on all levels (physical and non-physical), sensitive to authentic energy and consciousness, in which totality, beauty, comfort, dignity and peace are enhanced^(8,9)^.

The contributions of knowledge from nursing and other disciplines arise from multiple sources that were accessed through the integrative approach used in theoretical construction, using research processes based on deduction and induction reasoning. These reasonings were made compatible through the combination and aggregation of different data sources^(24)^ so that deduction was based on the Theory of Human Caring^(9)^ and the results of a synthesis of concepts^(3)^ that took into account consideration of knowledge about PC and gerontology.

The inductive strategy began with the development of an educational curriculum that aimed to improve the effectiveness of nurses’ practice in caring for older adults in their last days of life. This educational program was designed based on the focus group technique with nurses from an evidence-based nursing residency program in geropalliative care linked to a teaching hospital in the United States of America^(15)^.

It is important to highlight that the education program, a reflective practice based on evidence and guided by the Geropalliative Caring Model, developed in a teaching hospital^(15)^, was called AgeWISE and disseminated in 14 hospitals with the aim of improving nurses’ skills in PC as a basic competence of practice^(8)^.

The AgeWISE curriculum framework was developed not as a prescriptive curriculum, but as a portfolio of easily customizable learning activities to guide each site team in their implementation. The curricular matrix focused on eight fields to address PC: 1) structures and care field processes; 2) physical field; 3) psychological and emotional field; 4) social field; 5) spiritual, religious and existential field; 6) cultural field; 7) end-of-life care field; and 8) mastery of legislation and ethics field^(16)^.

Regarding the metaparadigmatic concepts of nursing used by Fawcett^(14)^, such as health, nursing, environment and person, we did not see a clear distinction from the more general content of SST. However, it is possible to identify them based on inferences based on the understanding of the Geropalliative Caring Model in relation to the concepts of human being, environment, health and nursing, following the recommendations of the analysis method that recommends that, when a theory’s author is not clear on a certain point or does not present some information, it may be necessary to make inferences or resort to other theory revisions.

Thus, it is possible to infer that “human being” is seen in its entirety as mind/body/spirit/universe, and the Geropalliative Caring Model focuses on the person and their family as the unit of care so that values, needs and preferences are guidelines for care planning^(2)^. Thus, the nursing team is expected to ensure conditions so that older adults and their families do not feel alone or abandoned^(8)^.

The “environment” must portray a space capable of promoting sensitive and responsive care to the needs of older adults in end-of-life care and their families^(15)^, based on nursing teams’ specific skills, authentic energy and awareness, with full attention, sensitive listening and true presence in a therapeutic environment (healing) in all dimensions of care, in addition to due attention to patient safety measures^(8)^.

The nursing and health concepts are intertwined with the purpose of promoting well-being to persons. Therefore, “nursing” aims to promote conditions that favor protection, promoting and restoring health and well-being as well as preventing illness and injury and alleviating the suffering of older adults in end-of-life care and their families. Well-being is the expected result of the nursing team’s actions.

The nursing team must advocate for the person/family using their clinical nursing knowledge and communication skills, ethical and moral principles to defend older adults when others (professionals or family members) choose to proceed with a treatment different from that which older adults desire or when others attempt to refuse the treatment that they have chosen for themselves^(8)^. In this way, nurses constitute a mediation platform between patient/family and the PC team given their privileged position of proximity to the care unit, making it possible to advocate for patients and encouraging them to fight for the exercise of their autonomy^(25)^.

Regarding “health”, its essential objective is also to promote ethical-moral-philosophical well-being consistent with the philosophical principles of PC^(1,2)^. Therefore, when restoration of health is not possible, given the severity of the terminal illness, well-being must be the focus of end-of-life care actions^(8)^ to be developed by the nursing team towards older adults and their families in order to alleviate suffering and promote comfort and human dignity.

The theorist explored the Geropalliative Caring Model content by synthesizing the concept of “geropalliative care”^(3)^.

Concept synthesis is a strategy for developing concepts based on observation or the search for empirical evidence, given that concepts can be developed from observation, clinical experience, collection of qualitative and quantitative data or a combination of them. It is worth noting that concept synthesis differs from concept analysis, as the former is useful when few or no concepts are available or when attributes are unknown. The latter is convenient when the concept is already available in the area of interest^(26)^.

Lee^(3)^ identified the singularities between PC and gerontology, considering that both fields of knowledge emphasize the importance of working in an interdisciplinary team, patient- and family-centered care, alleviating suffering and seeking to provide better quality of life for people. In the case in question, at the time, the first decade of the 21^st^ century, the concept did not yet have its attributes clear.

From the application of concept synthesis, it was possible to infer that geropalliative care represents more than the sum of geriatric care and PC, highlighting that all the philosophical principles of PC apply to the care of older adults. However, it was possible to relate the essential attributes of PC with the specificity of older adults so that it is possible to promote safe and effective care^(3)^. This concept has nine critical attributes, namely: 1) high risk for ineffective pain control; 2) unpredictable disease trajectory; 3) geriatric syndromes (frailty, polypharmacy and altered pharmacokinetics, dementia, delirium and unusual clinical conditions); 4) chronic conditions and comorbidities; 5) decrease in social support networks; 6) limitations of health insurance; 7) multiple care scenarios/contexts; 8) risk of ineffective communication; and 9) benefit only due to age, regardless of the disease^(3,13)^.

Based on such critical attributes, it is possible to affirm that geropalliative care is both a philosophical stance^(1,2)^ and a structured and interdisciplinary model of care provision that guides care planning for patients and families in their last five years of life, regardless of whether they have a disease or not^(3)^.

**Figure 1.**
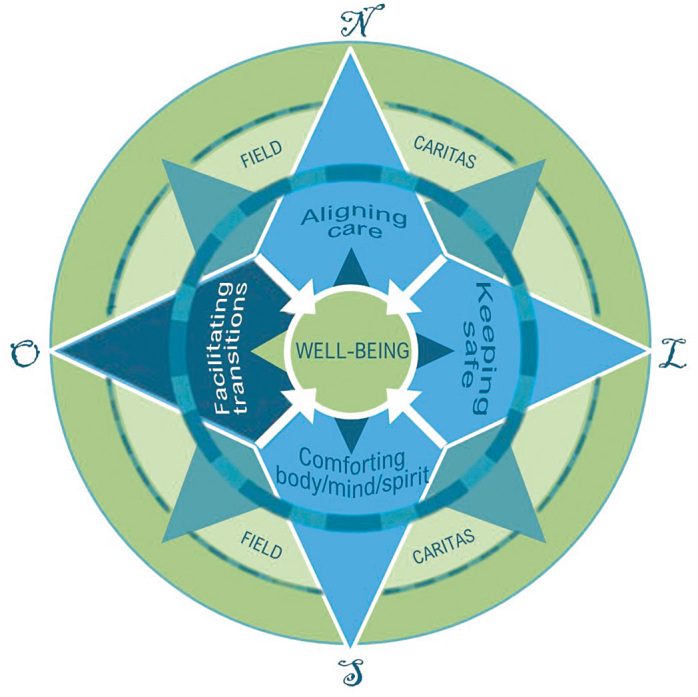
Lee Geropalliative Caring Model^(8,13)^.

Based on these attributes, it was possible to structure the Geropalliative Caring Model based on four fields: 1) aligning care; 2) keeping safe; 3) comforting body/mind/spirit; and 4) facilitating transitions (Figure 1). Each field correlates with the four principles of bioethics, such as autonomy, nonmaleficence, beneficence and justice, respectively^(8)^.

Geropaliative care encompasses philosophical principles and moral values that are central to PC, recognizing that nonmaleficence and beneficence require specific knowledge of age-related phenomena to optimize care of older adults in the last years of life and their families. Thus, the scope of geropalliative care is to promote the well-being of older adults and their families through interventions that reduce suffering and improve quality of life^(3,8)^.

A striking concept in the model is “transition”, which is closely related to concepts of health and well-being, involving psychological and emotional processes in which older adults and their families need time and support to adapt to a changing reality^(27)^. The theorist recognizes that transition is, therefore, a fundamental concern in nursing discipline, regardless of the specialty, as it involves processes of movement from one state, condition or situation to another, in this context, from a healthy life to living with a chronic and life-threatening disease, which demands a PC approach^(3,8)^.

The concept of transition underlies the fourth field of the model, which is characterized by acts of helping people/families in transition to a life condition marked by a chronic illness or changes in older adults’ functional status and even to a dignified death^(3,8,27)^.

Theory assessment requires the interpretation of the Geropalliative Caring Model significance, internal consistency, parsimony, testability, empirical adequacy and pragmatic adequacy criteria^(14)^.

As for their significance, which requires justification of the theory for the discipline, they are explicit, clearly integrated with the theory’s central concepts, represented by four fields: 1) aligning care; 2) keeping safe; 3) comforting body/mind/spirit; and 4) facilitating transitions. This is significant for using the theory in favor of the well-being of older adults in end-of-life care and their families. Furthermore, its development is based on the justification that there is a need for PC for older adults, which is characterized by particularities related to the aging process, according to the aforementioned nine critical attributes that differentiate PC for older adults from other phases of human development^(3)^.

There is also significance to training. The theorist highlights contributions of theory to improving nurse training curriculum of an evidence-based nursing residency program in geropalliative care^(15)^, which was replicated as an AgeWISE education program^(16)^ in 14 hospitals in the United States to improve nurses’ skills in gerontological PC.

The Geropalliative Caring Model’s internal consistency is linked to theoretical content and context. We verified semantic consistency in SST concepts, as well as its structural consistency is based on the four fields and their interrelationship with the bioethical principles of autonomy, nonmaleficence, beneficence and justice. There is relevance in the central premise in the search for the well-being of older adults in end-of-life care and their families, promoting a therapeutic (healing) and caring environment, respecting older adults’ preferences, alleviating their suffering and encouraging comfort measures in for a dignified death^(8)^. Moreover, the theory content indicates that it would be up to nurses to provide discussions and anticipatory guidance that make it possible to accommodate patients’ demands and guide the family in decision-making^(8)^.

Still in relation to internal consistency according to Fawcett’s assessment criteria^(14)^, we believe that SST has clarity, conciseness and articulation between the Geropalliative Caring Model fields in favor of the well-being of older adults in end-of-life care and their families, which reflects the theory’s parsimony criterion, in addition to being in line with PC philosophical principles pre-established by the World Health Organization^(1,2)^.

The theory’s testability criterion was assessed based on the consideration that articulation of SST concepts supported the construction and application of a curriculum for gerontology nurses specializing in PC in a residency program that trained 108 nurses^(16)^. Thus, a commitment is made to promote measures for high-quality nursing care based on acquired skills, indicating that geropalliative care has the potential to promote care focused on alleviating suffering and improving the quality of life of older adults who live with chronic diseases (Alzheimer’s disease, heart/liver/kidney failure, cancer, neurological diseases, among others) and/or frailty and their families^(16)^.

For the empirical and pragmatic adequacy criteria, there is still potential to advance the verification of new data on the practical use of SST as its application to other realities occurs over the years. In addition to the theory’s testability using the AgeWISE program^(16)^, replication in other settings is necessary, including in different cultures. It is also essential to expand studies on the theory’s practical applicability, in order to allow their results to generate new scientific evidence that will support research such as meta-analysis or meta-synthesis in order to integrate the results of related studies^(14)^.

The analysis and assessment of the Geropalliative Caring Model carried out in this article have some contributions that can help a user of the theory who is not experienced in metatheoretical knowledge. Initially, a mid-range nursing or SST theory must have elements to guide professional practice, and some questions can be applied for this purpose: is there a need for additional studies? What still needs to be done? What can be tried and what should not be done^(14)^?

When using empirical and pragmatic adequacy criteria, it was established that additional studies need to be carried out to verify good or bad results from the use of SST in care situations beyond those already documented in the literature. For instance, conjectural extrapolations may indicate that the theory may not be fully accepted in strongly functionalist contexts, where care is not person-centered and humanistic criteria on which Lee’s theory was based.

Every theory needs to be continually updated as practical situations indicate limits in its application. Therefore, tests with field research can employ working hypotheses drawn from the Geropalliative Caring Model propositions and concepts. This ultimately reinforces security in good virtues of the theory.

The creation of new products linked to theory can be attempted as a strategy that would advance theoretical development. Guidelines, protocols and scales supported by the Geropalliative Caring Model can be created, ensuring that elements of theory can more easily translate into clinical practice.

Explicitly, what should not be done is to assume that a theory is irrefutable or has no limitations. Although it is not common for theorists to point out the limitations, flaws or problems of their “work”, such aspects exist. The analysis and assessment carried out in this theoretical study indicated some limitations of the theory in what could be verified in the literature and in the interpretations made about such publications. However, it is in the space of practical operationalization and research that a theory proves useful, which justifies the continued advancement of research into aspects related to the Geropalliative Caring Model, mainly experiences of use in care.

A limitation of this study was that the analysis and assessment carried out were not able to identify the meaning of the terms “model” and “theory” that guided the theorist in SST construction. We found that Lee sometimes uses the term interchangeably, making it difficult to define its limits of use in a metatheoretical view. Although there are perspectives that consider “models as theories”^(28)^, it was not possible to verify whether this is in fact the case.

## FINAL CONSIDERATIONS

The study made it possible to assess the Geropalliative Caring Model based on the steps of theory analysis and assessment according to Fawcett, highlighting that it is a theory that was developed based on the theorist’s professional trajectory when the structuring of a curriculum for training residents in gerontological nursing, which made it possible to understand that the theory has gerontological care as its scope from a philosophical perspective of PC, taking into account the specificities of the aging process.

The analysis of results demonstrated that the theorist presents it as an SST, with well-being as the central concept. It was derived from a grand theory, the Theory of Human Caring, in which the Clinical Caritas Process, composed of 10 elements, is interconnected considering its level of abstraction with the four fields of the Geropalliative Caring Model: aligning care; keeping safe; comforting body/mind/spirit; and facilitating transitions. The concept of transition was recognized as a fundamental concern of the nursing team, as it is a condition that involves changes in the lives of both older adults undergoing end-of-life care and the family who needs to learn to deal with the process of dying and death of their loved ones.

It also made it possible to incorporate the description of the four concepts of nursing metaparadigms, having at its core the care for the well-being of older adults in end-of-life care based on bioethics principles, such as autonomy, nonmaleficence, beneficence and justice, which are interconnected with the four fields of the theory.

The presumed contributions of this analysis and assessment research are to expand nursing students’ and professionals’ knowledge about SST, encouraging reflections on the specificities of older adults and the PC context itself, influencing professional practice through care actions for the well-being and a dignified death of these people in end-of-life care, and, finally, offering the opportunity for normal mourning for their families.

We recognize that further studies, especially on pragmatic testing and assessment, are welcome. The systematic application of theory in different contexts can generate new evidence that allows robust studies that expand theoretical adequacy assessment.
